# The Impact of Nutrition on the Onset, Course of Disease and Quality of Life of Patients with Laryngopharyngeal Reflux

**DOI:** 10.17113/ftb.61.04.23.8222

**Published:** 2023-12

**Authors:** Tin Prpić, Melita Peček Prpić, Tihana Mendeš, Anamarija Šestak, Andrijana Včeva

**Affiliations:** 1 J.J. Strossmayer University of Osijek, Faculty of Medicine in Osijek, Josipa Huttlera 4, 31000 Osijek, Croatia; 2 Clinical Medical Center Osijek, Josipa Huttlera 4, 31000 Osijek, Croatia; 3 Osijek Health Center, Park kralja Petra Krešimira IV 6, 31000 Osijek, Croatia

**Keywords:** laryngopharyngeal reflux, food with low reflux potential, quality of life, dietary habits

## Abstract

**Research background:**

The role of dietary habits of patients with laryngopharyngeal reflux (LPR) is comparatively underexplored. The aim of the study is to examine dietary habits, onset and course of the disease as well as the quality of life of patients with LPR.

**Experimental approach:**

The results of the modified food frequency questionnaire (FFQ-m) and laryngopharyngeal reflux health-related quality of life (LPR-HRQL) questionnaires were compared between subjects with and without LPR. There were a total of 100 subjects with LPR and 65 subjects in the control group. The group of subjects with LPR was further randomly divided into two subgroups; the first subgroup was treated with esomeprazole at a dose of 20 mg twice daily combined with the instructions for dietary and general lifestyle changes, and the other with pantoprazole at a dose of 20 mg twice daily combined with the instructions for dietary and general lifestyle changes. Participants were instructed to fill out FFQ-m and LPR-HRQL questionnaires immediately after the initial examination and then after control examinations 30 and 60 days after the initial examination.

**Results and conclusions:**

Patients with LPR consume more food with high reflux potential, drink more carbonated drinks and juices and have a worse quality of life than the control group (p<0.001). Taking proton pump inhibitors at a dose of 20 mg twice daily in combination with a change in dietary habits such as substituting acidic, spicy, fermented, sweet, fried foods and other foods with a high reflux potential as well as carbonated drinks and juices with the food with a low reflux potential and water significantly reduced the symptoms of LPR and increased the quality of life of the patients (p<0.001).

**Novelty and scientific contribution:**

This is the first study showing the correlation between dietary habits and the quality of life of patients with LPR. The contribution of this research is an objective assessment of the follow-up of patients with LPR that could be used in their regular assessment.

## INTRODUCTION

Laryngopharyngeal reflux (LPR) is a clinical condition that represents the return of gastric contents into the space of the larynx and hypopharynx, where it makes close contact with the tissues of the upper aerodigestive tract (*1*). Two theories explain the pathogenesis of reflux laryngitis. The first is the theory of direct injury to the mucosa of the larynx and surrounding tissue by acid and pepsin. This results in damage to the mucociliary transport and accumulation of secretions in the throat, which causes additional irritation of the mucosa and contributes to the onset of symptoms of laryngopharyngeal reflux. Namely, the larynx does not have protective external cleaning mechanisms and saliva cover that neutralise acids, so the gastric refluxate remains undiluted for a longer period of time, resulting in tissue injury. The second theory that explains the pathogenesis of reflux laryngitis is the reflex theory. According to this theory, LPR occurs due to oesophageal reflux, which stimulates vagally mediated reflexes resulting in chronic throat clearing and coughing that leads to mucosal injury of the larynx (*2*, *3*). Nine of the most common symptoms of LPR were quantified by Belafsky *et al.* (*4*) in the so-called reflux symptom index (RSI), and based on years of experience, he concluded that if the RSI is greater than 13, LPR can be suspected. It is necessary to score the severity of the symptoms using a scale of 0–5 (0 meaning no symptoms to 5 meaning severe symptoms). The most common symptoms of LPR are hoarseness, throat clearing, postnasal drip, swallowing difficulties, coughing after meals or upon lying down, feeling of choking, coughing attack, globus sensation, heartburn and chest pain. Belafsky *et al.* (*4*) quantified the eight most common clinical signs of LPR in the reflux finding score (RFS). If the RFS is greater than 7, suspicion of LPR can be raised. The doctor calculates the RFS based on the presence or absence and severity of clinical signs of LPR. Some of the most common clinical signs of the disease are vocal fold oedema, granulation tissue, laryngeal posterior commissure hypertrophy, erythema, subglottic oedema, diffuse laryngeal oedema, ventricular obliteration and thick endolaryngeal secretion (*5*). Considering the severity of the symptoms and the impact on the quality of life of the patients, LPR can be mild, severe or life-threatening. Mild LPR bothers patients but does not interfere with their daily activities. Severe LPR significantly impairs the quality of life and interferes with patients’ daily tasks and personal activities. Life-threatening LPR is present in patients with airway obstruction (*6*, *7*).

There is no gold standard for the treatment of LPR. It is treated with changes in diet, lifestyle and drugs such as proton pump inhibitors (*8*). Dietary measures include avoiding tea, coffee, fatty and spicy foods, alcohol, chocolate and carbonated drinks. The intake of alkaline foods such as bananas and melons is recommended. As for drinks, only plain or alkaline water is recommended. Furthermore, food should be consumed in smaller portions, more frequently and should not be taken within two hours of bedtime. It is necessary to raise the head of the bed when lying down, and one should not lie down immediately after eating. If the person is a smoker, he or she must quit smoking (*3*, *9*). In addition to conservative treatment, surgical procedures such as transoral fundoplication and magnetic sphincter augmentation (*3*, *10*) can be performed on patients who are refractory to conservative therapy.

It is known that lifestyle and dietary habits, such as smoking, consumption of alcohol and of acidic, sweet and spicy foods play a significant role in the development of LPR. The food frequency questionnaire is commonly used to assess dietary habits in clinical studies and it estimates the frequency of consumption of beverages and foods (*11*, *12*). Lifestyle habits, including eating habits, vary in different countries and are often culturally conditioned (*13*, *14*). Therefore, eating habits should be studied separately for each region. Furthermore, the role of eating habits of patients with laryngopharyngeal reflux disease has been comparatively scarcely researched. In our study, we aim to investigate eating habits and their influence on the onset, course of disease, and the quality of life of patients with LPR in eastern Croatia.

## MATERIALS AND METHODS

### Study design

The study was designed as a controlled non-randomised clinical trial with the aim to investigate the influence of diet on the onset, course of disease and the quality of life of patients with LPR. Participants were divided into two main groups: patients with LPR and a control group without LPR. Each participant underwent a comprehensive otorhinolaryngological examination followed by laryngeal endoscopy.

The first group consisted of participants who suffered from LPR. The diagnosis of LPR was based on RSI (reflux symptom index) and RFS (reflux finding score). Patients with RSI scores greater than 13 and RFS greater than 7 were included in the group of LPR patients. Patients with RSI scores lower than or equal to 13 and RFS lower than or equal to 7 were included in the control, healthy group.

The LPR group was randomly divided into two subgroups according to the type of proton pump inhibitor (esomeprazole or pantoprazole) using a remote computer-generated code. The first subgroup was treated with esomeprazole at a dose of 20 mg twice daily and the second subgroup was treated with pantoprazole at a dose of 20 mg twice daily. Both subgroups received written instructions on dietary and general lifestyle changes and adhered to the therapy and instructions for 60 days.

The general lifestyle instructions included eating small portions, managing stress, sleeping with an elevated headrest, avoiding eating before bedtime, quitting smoking and avoiding caffeine consumption before bedtime. All participants documented their daily food intake from the initial examination to the last follow-up examination. In addition, instructions were given on how to record daily food intake. Based on the daily food intake diary, the food intake of all patients was evaluated using the refluxogenic diet score developed by Lechien *et al*. (*15*). The refluxogenic diet score is based on the pH value of the food and its composition. Based on the final score, all foods are categorised into one of five categories according to their refluxogenic potential. In line with the results of the refluxogenic diet score, we categorise foods into those with low and high refluxogenic potential. Therefore, dietary instructions also included recommendations for consumption of foods with low refluxogenic potential according to the refluxogenic diet score, such as corn, rice, oatmeal, melons, watermelon, carrots, lettuce and cereals, and avoiding foods with high refluxogenic potential such as yogurt, pears, apples, oranges, grapefruit, mandarins, nectarines, peaches, bacon, butter and cookies. Additionally, each participant was required to complete a modified food frequency questionnaire (FFQ-m) and laryngopharyngeal reflux health-related quality of life (LPR-HRQL) questionnaires immediately after the initial examination and after the follow-up examinations, which were conducted 30 and 60 days after the initial examination.

### Participants

The study included adult patients with LPR who responded positively to the invitation to participate in it at the Clinic of Otorhinolaryngology and Head and Neck Surgery at the Clinical Hospital Centre Osijek, Croatia. The control group consisted of healthy adult participants who did not have LPR and were scheduled for surgery for otapostasis, deviated septum, nasal polyps or were employees of the Clinical Hospital Centre Osijek and agreed to participate in the study. Participants were informed about the study and their written consent was obtained. The study was approved by the Ethics Committee of the School of Medicine, University of Osijek (UR number: 2158-61-46-22-39).

Recruitment was conducted until a total of 200 participants was reached. Exclusion criteria were gastrointestinal ulcer disease, chronic atrophic gastritis, cancers and treatment with proton pump inhibitors, antacids or H2 blockers. Additionally, participants who did not correctly complete all the questionnaires were excluded from the analysis. Thirty-five participants did not meet the inclusion criteria. Therefore, 165 participants were included in the study, with 100 (60.6 %) participants in the LPR group and 65 (39.4 %) participants in the control group. There were a total of 69 (41.8 %) men and 96 (58.2 %) women. The median age of all participants was 49 (interquartile age range 18 to 82). In the group of participants with LPR, 36 (51 %) received pantoprazole therapy and 34 (49 %) received esomeprazole therapy. In our study, the LPR-HRQL questionnaire was used to assess the quality of life of patients with LPR and the control group as the treatment outcome.

### FFQ-m questionnaire

FFQ-m questionnaire is a tool used to estimate the frequency of consumption of foods and beverages in the last month (*16*). A modified questionnaire was used based on an existing one developed and validated in Croatia by Močić Pavić *et al.* (*17*). The questionnaire was modified by regrouping certain food groups and only assessing the frequency of consumption of a particular food item, without evaluating the portion size. The modified FFQ contained 75 different food and beverage items divided into 12 different groups. The food categories were: (*i*) fast food, (*ii*) milk and dairy products, (*iii*) milk and dairy products with added sugar, (*iv*) fats and oils, (*v*) cereals, (*vi*) salty snacks, sweets and cakes, (*vii*) breakfast cereals, (*viii*) processed meat, (*ix*) juices, (*x*) vegetables, (*xi*) fruit and (*xii*) meat, fish and eggs. The frequency of food consumption is scored in the range from 0 to 8, where 0 means never, 1 refers to one to three times a month, 2 to once every week, 3 to two to four times a week, 4 to five to six times a week, 5 to once a day, 6 to two to three times a day, 7 to four to five times a day and 8 refers to six or more times a day (*17*).

### LPR-HRQL questionnaire

The LPR-HRQL questionnaire consists of 43 questions that assess how often or to what extent the respondent experiences certain feelings. The first 12 questions relate to speaking, singing and voice and, together with the 13th question on the impact of voice on quality of life, form the voice/hoarse section. The cough section consists of questions 14-19, which assess coughing, and the 20th question on the effect of coughing on quality of life. The clear throat section consists of questions 21-26, which assess throat clearing, and question 27 on the effects of throat clearing on quality of life. Questions 28-32 relate to swallowing and general throat-related symptoms, with the 33rd question on the effect of swallowing on quality of life, thus covering the swallow section. Finally, questions 34-43 assess the effect of acid reflux symptoms on quality of life and form the section of the overall impact of the acid reflux. A standard Likert scale ranging from 0 to 6 was used, with a higher score indicating more frequent LPR symptoms, *i.e*. 0 refers to never, 1 to once a month, 2 to two to three days a month, 3 to one day a week, 4 to two to three days a week, 5 to four to five days a week and 6 refers to six to seven days a week. The score for the last question in each section, as well as all 10 questions in the section overall impact of acid reflux, range from 1 (no effect) to 10 (enormous effect on health-related quality of life) (*18*, *19*).

### Statistical analysis

Categorical data are represented by absolute and relative frequencies. The normality of the distribution of numerical variables was tested with the Shapiro-Wilk test. Numerical data are described by the median and the limits of the interquartile range. Differences between two independent groups were tested with the Mann-Whitney U test (95 % difference in confidence interval), and differences between measurements with the Friedman test (*post hoc* Conover). All p-values were two-sided. The significance level was set at α=0.05. The statistical analysis was performed using MedCalc® statistical software, v. 20.218 and SPSS, v. 23 (*20*, *21*).

## RESULTS AND DISCUSSION

In addition to pantoprazole and esomeprazole therapy, other proton pump inhibitors such as omeprazole, lansoprazole and rabeprazole have often been used in other studies. The success rate in treating LPR symptoms with these proton pump inhibitors ranges from 18 to 87 % without significant differences in treatment outcomes based on the therapy used (*4*, *22*, *23*). Comparable to the results of these studies and considering the treatment outcome through subjective assessment of quality of life using the LPR-HRQL questionnaire, in our study there was no significant difference in any of the sections of the LPR-HRQL questionnaire between patients who used esomeprazole and pantoprazole after 30 and 60 days of therapy. Additionally, the median value of the overall impact of acid reflux was lower after 30 and 60 days in both mentioned groups than the value of the initial examination (Table 1).

**Table 1:** Differences in the LPR-HRQL scale based on the applied therapy on group of patients with LPR symptoms measured at three different times

Symptom	Median (interquartile range)		Difference (95 % confidence interval)	p*
Pantoprazol	Esomeprazol
*t*=0 day				
Voice/hoarseness	22 (14–28)	16 (9–22)	-5 (-9–0)	0.03
Cough	11 (9–14)	9 (6–11)	-3 (-5–0)	0.06
Throat clearing	11 (7–14)	8 (5–16)	-2 (-4–2)	0.33
Swallowing	7 (4–11)	8 (6–12)	1 (-2–3)	0.48
The overall impact of acid reflux	36 (29–47)	34 (28–40)	-3 (-10–3)	0.34
*t*=30 days				
Voice/hoarseness	14 (9–22)	11 (7–19)	-2 (-7–2)	0.25
Cough	6 (2–10)	3 (0–9)	-2 (-5–0)	0.09
Throat clearing	6 (2–10)	4 (1–6)	-1 (-3–1)	0.38
Swallowing	4 (2–9)	6 (3–10)	1 (-1–3)	0.44
The overall impact of acid reflux	31 (15–40)	28 (22–39)	-1 (-8–7)	0.78
*t*=60 days				
Voice/hoarseness	11 (9–14)	9 (6–11)	-3 (-5–0)	0.05
Cough	2 (1–7)	3 (0–4)	0 (-2–1)	0.61
Throat clearing	2 (1–5)	3 (1–5)	0 (-1–2)	0.83
Swallowing	3 (2–6)	3 (2–6)	0 (-2–2)	0.88
The overall impact of acid reflux	20 (13–25)	20 (14–29)	0 (-6–6)	0.90

*Mann Whitney U test, LPR-HRQL=laryngopharyngeal reflux health-related quality of life

In the group of patients with LPR, there were significantly higher values (more frequent occurrence of symptoms, p<0.001) in all sections of the LPR-HRQL questionnaire at the initial examination than in the control group. It should also be noted that the most common symptom in the group of patients with LPR was hoarseness (Table 2). Patients with LPR had similar median values in all domains of the LPR-HRQL questionnaire to the values reported by other authors (*18*, *19*). However, small differences were found between our study and a Swedish study (*18*), in which the results showed lower values ​​of all LPR-HRQL sections than in our study, which could be explained by different cultural settings and differences in dietary habits.

**Table 2:** The difference in individual domains of the LPR-HRQL scale compared to the groups at the initial examination (*t*=0)

Symptom	Median (interquartile range)		Difference (95 % confidence interval)	p*
Control group	Group with LPR
Voice/hoarseness	5 (4–7)	19 (11–26)	10 (8–14)	<0.001
Cough	0 (0–3)	8 (3–12)	6 (4–7)	<0.001
Throat clearing	0 (0–2)	9 (5–14)	8 (6–9)	<0.001
Swallowing	0 (0–3)	7 (4–11)	6 (4–7)	<0.001
The overall impact of acid reflux	10 (9–20)	32 (24–43)	19 (15–22)	<0.001

*Mann Whitney's U test, LPR-HRQL=laryngopharyngeal reflux health-related quality of life

In the group of patients with LPR, the values ​​of all LPR-HRQL sections were significantly lower (p<0.001) after 60 days than the initial values (Table 3). Therefore, we can say that the recommended therapy with proton pump inhibitors along with instructions on diet and lifestyle changes was definitely successful. In their study, Carrau *et al.* (*19*) measured the LPR-HRQL at baseline, after 4 and 6 months, and the therapy used was a dose of 20 mg omeprazole twice a day. Our study shows a similar improvement in scores between the pretreatment and post-treatment status compared to Carrau's study, although the measurements were taken after 4 and 8 weeks. Furthermore, in our study, patients were given dietary and general lifestyle instructions in addition to the proton pump inhibitors as part of the treatment. Several studies using the LPR-HRQL and surgical procedures such as fundoplication for LPR treatment have also shown a significant improvement in scores before and after the procedure (*24*, *25*).

**Table 3:** Ratings of LPR-HRQL questionnaire in a group of patients with LPR symptoms at three measured times

Symptom	Median (interquartile range)			p*
*t*/day		
10	30	60
Voice/hoarseness	19 (11–26)	13 (8–21)	10 (7–13)	<0.001^†^
Cough	8 (3–12)	4 (1–9)	2 (0–6)	<0.001^†^
Throat clearing	9 (5–14)	5 (2–10)	2 (1–5)	<0.001^†^
Swallowing	7 (4–11)	7 (3–10)	3 (2–6)	<0.001^†^
The overall impact of acid reflux	32 (24–43)	31 (18–40)	20 (14–26)	<0.001^†^

*Frideman's test (*post hoc* Conover), ^†^significantly different values at all three measured times (p<0.05), LPR-HRQL=laryngopharyngeal reflux health-related quality of life

We examined the dietary habits of participants with LPR and the control group using a modified food frequency questionnaire (FFQ). Compared to patients with LPR at the beginning of the research, the control group consumed significantly more frequently pudding, semolina, polenta, rice, corn (cooked/baked), cornflakes, oatmeal, muesli, sugar-free soft drinks, bananas, melons, watermelon, carrots, spinach, chard and lettuce. The group of patients with LPR symptoms consumed significantly more frequently sour cream (12 % fat), yogurt, acidophilus milk, kefir (2.8 to 3.2 % fat), fruit yogurt, white bread (pastries and puff pastry), carbonated soft drinks, fruit syrups (fruit concentrate), apples or pears, oranges, grapefruits, mandarins, peaches, nectarines, grapes, onions, garlic, breaded pork, bacon, cookies, margarine, oils and hamburgers (Table 4). According to the results, patients with LPR consumed significantly more frequently fatty, fermented, sweet, and acidic foods and acidic drinks, which can increase the number of proximal reflux episodes and are important risk factors for developing LPR (*8*, *26*). Lechien *et al.* (*15*) developed the refluxogenic diet score as an objective assessment of the refluxogenic potential of the food. In our study, participants without symptoms of LPR consumed more food with low refluxogenic potential (corn, rice, oatmeal, bananas, melons, watermelon, carrots, lettuce and cereals), while participants with LPR symptoms consumed significantly more frequently food classified as high refluxogenic food according to the refluxogenic diet score (yogurt, pears, apples, oranges, grapefruits, mandarins, nectarines, peaches, bacon, pork, butter and cookies) (Table 4).

**Table 4:** Differences in the frequency of consumption of individual food items (divided in food categories according to modified FFQ) at initial examination (*t*=0) with respect to the control and LPR group (Mann Whitney’s U test)

Food	Median (interquartile range)		p
Control group	Group with LPR
Milk and dairy products			
Cream (*w*(milk fat)=12 %)	2 (1–2)	3 (1–4)	<0.001
Yogurt, acidophilus, kefir (*w*(milk fat)=2.8–3.2 %)	2 (1–3)	3 (1–4)	<0.001
Fruit yogurt	1 (1–2)	2 (1–3)	0.02
Pudding	2 (1–3)	2 (0–2)	0.02
Cereals			
White bread (rolls and croissants)	3 (1–4)	5 (3–5)	<0.001
Semolina	2 (1–3)	1 (0–2)	<0.001
Polenta	2 (1–3)	1 (0–2)	<0.001
Rice	3 (2–4)	2.5 (1–3)	0.03
Corn (cooked/roasted)	2 (2–3)	1 (0–2)	<0.001
Breakfast cereals			
Cornflakes	2 (1–3)	1 (0–2)	<0.001
Oatmeal and muesli	2 (1–2)	1 (0–2)	<0.001
Beverages			
Orange juice	2 (2–3)	3 (2–4)	0.01
Carbonated soft drinks	2 (1–3)	3 (2–4)	<0.001
Non-alcoholic drinks (sugar-free)	6 (5–7)	4.5 (3–5)	<0.001
Fruit syrup (syrup concentrate)	2 (0–2)	2 (1–3)	<0.001
Fruit			
Apple or pear	2 (1–3)	3 (2–4)	0.01
Orange, grapefruit, tangerine	2 (1–3)	3 (1.25–4)	<0.001
Banana	3 (2–3.5)	2 (1–3)	<0.001
Peach, nectarine	1 (0–2)	2 (0–3)	0.02
Melon, watermelon	2 (1–3)	1 (0–1)	<0.001
Grapes	1 (0–2)	2 (1–3)	<0.001
Vegetables			
Onion, garlic	2 (1–3)	3 (2–4)	<0.001
Carrot	2 (1.5–3)	2 (1–3)	<0.001
Spinach, chard	2 (2–3)	2 (1–2)	<0.001
Lettuce	3 (2–4)	3 (2–4)	<0.001
Meat, fish and eggs			
Breaded pork	2 (1–3)	2.5 (1–3)	<0.001
Processed meat			
Bacon	2 (2–3)	3 (2–3)	<0.001
Salty snacks, sweets and cakes			
Biscuits	2 (1–2)	2.5 (1–3)	<0.001
Fat and oil			
Margarine	2 (2–3.5)	3 (1–3.75)	0.02
Oil	3 (2–5)	4 (2–5)	0.03
Fast food			
Hamburger	1 (0–2)	2 (1–3)	<0.001

FFQ=food frequency questionnaire, LPR=laryngopharyngeal reflux

In addition to the proton pump inhibitors, patients with LPR received written instructions on diet and lifestyle changes. They documented their daily food intake and, according to the instructions, they were told which foods had low and which had high refluxogenic potential (*15*). The patients with LPR significantly reduced the frequency of consumption of a large number of foods considered highly refluxogenic according to the refluxogenic diet score (Table 5 and Table 6). Such a change in diet could have an effect on the reduction of LPR symptoms, *i.e.* a significant reduction in the values of all LPR-HRQL sections and an improvement in the quality of life, as can be seen in Table 3. It is very important to give patients clear instructions on how to change their lifestyle and diet, to encourage them to write down the foods they consume every day and, of course, to monitor and advise them regularly. In their analysis of LPR therapy, Runggaldier *et al.* (*27*) particularly emphasise the importance of diet, which they consider one of the key factors for the success of LPR treatment. The meta-analysis by Min *et al.* (*28*) showed that avoiding fatty foods, chocolate and coffee while maintaining a Mediterranean diet and consuming alkaline water significantly reduced the symptoms and clinical signs of LPR. However, although the emphasis is on lifestyle and diet changes, the patients in our study took proton pump inhibitors daily, which could have contributed to better LPR-HRQL results. The study by Yang *et al.* (*29*) is also of interest, in which they compared the treatment of LPR by dietary and lifestyle changes with medication treatment. As much as 95 % of participants in the group that accepted diet change reported subjective improvement of LPR symptoms after the treatment. On the other hand, in the group of participants treated solely with anti-reflux medications, only 48 % of participants reported an improvement.

**Table 5:** Differences in the frequency of consumption of certain food items (divided in the first six food categories according to modified FFQ) by group with LPR symptoms at three measured times (Friedman's test *post hoc* Conover)

Food	Median (interquartile range)			p
*t*/day		
0	30	60
Milk and dairy products				
Milk	3 (2–5)	2 (1–4)	2 (0–3)	0.01^†^
Fresh cottage cheese	2 (1–3)	2 (0–3)	1 (0–2)	0.005^‡^
Cream (*w*(milk fat)=12 %)	3 (1–4)	2 (0–3)	1 (0–3)	0.009^§^
Semi-hard and hard cheese	2 (1–3)	1 (1–2)	1 (0–2)	0.006^§^
Cheese spread (*w*(milk fat)=30 %)	2 (1–3)	2 (1–3)	1 (0–2)	0.003^‡^
Yogurt, acidophilus, kefir (*w*(milk fat)=2.8–3.2 %)	3 (1–4)	2 (1–3)	1 (0–2)	0.001^‡^
Milk and dairy products with added sugar				
Cocoa/chocolate milk	2 (0–2)	1 (0–2)	1 (0–1)	0.02^‡^
Fruit yogurt	2 (1–3)	2 (0–2)	1 (0–2)	0.02^‡^
Cereals				
White bread (rolls and croissants)	5 (3–5)	3 (2–5)	3 (0.5–4)	<0.001^‡^
Rye/wholemeal bread (rolls and croissants)	2 (0–3)	3 (1.75–4)	4 (3–5)	<0.001^‡^
Polenta	1 (0–1)	1 (0–2)	2 (1–3)	<0.001^║^
Breakfast cereals				
Chocolate wheat flakes	1 (0–2)	2 (0–3)	1 (0–2)	0.006**
Cornflakes	0 (0–2)	1 (0–3)	1.5 (0–2)	0.02^††^
Beverages				
Orange juice	3 (2–4)	2 (0–3)	1 (0–2)	<0.001^‡‡^
Other juices	3 (2–4)	1 (0–3)	1 (0–2)	<0.001^‡‡^
Fruit juice	2 (1–3.75)	1 (0–2.25)	1 (0–2)	0.007^†^
Sweet vitamin drink	2 (1–3.75)	1.5 (0–3)	0 (0–2)	0.005^‡^
Ice tea	2 (1–3)	1 (0–2)	0 (0–2)	<0.001^†^
Carbonated soft drinks	3 (2–4)	2 (0–4)	1 (0–2.5)	<0.001^‡‡^
Non-alcoholic drinks (sugar-free)	4.5 (3–5)	5 (4–6)	6 (5–7)	<0.001^‡‡^
Fruit syrup (syrup concentrate)	2 (1–3)	2 (0–3)	1 (0–2)	0.002^‡^
Fruit				
Apple or pear	3 (2–4)	2 (0–3)	2 (0–2)	<0.001^‡‡^
Orange, grapefruit, tangerine	3 (1.25–4)	1 (0–2)	0 (0–1)	<0.001^‡‡^
Banana	2 (1–3)	3 (1–4)	3 (2–4)	<0.001^‡‡^
Melon, watermelon	1 (0–1)	2 (0–3)	2 (1–3)	<0.001^‡‡^
Grape	2 (1–3)	1 (0–3)	0 (0–1.5)	<0.001^‡‡^
Pineapple	0 (0–1)	1 (0–2)	1 (0–1.25)	0.009*

^†^significantly most consumed at *t*=0 day, ^‡^significantly least consumed after *t*=60 day, ^§^significantly less consumed after *t*=60 day than at *t*=0 day, *significantly least consumed at *t*=0 day, **significantly more consumed after *t*=30 day than after *t*=60 day, ^††^significantly less consumed at *t*=0 day than after *t*=30 day, ^‡‡^frequency of consumption is significantly different between all measurements, p<0.05

**Table 6:** Differences in the frequency of consumption of certain food items (divided in another six food categories according to modified FFQ; continuation of Table 5) by group with LPR symptoms at three measured times (Friedman's test *post hoc* Conover)

Food	Median (interquartile range)					p
*t*/day				
0	30		60	
Vegetables						
Onion, garlic	3 (2–4)		2 (1–4)		2 (1–2.75)	<0.001^‡‡^
Peppers (fresh and sauces)	3 (2–4)		2 (1–3)		1 (0–2)	0.002^§^
Tomato (fresh, sauces and salsas)	3 (2–4)		2 (1–2)		1 (0–1.5)	<0.001^‡‡^
Cabbage, kale	2 (1–3)		1 (1–2.25)		1 (0–2)	0.002^§^
Lettuce	3 (2–4)		2 (1–2)		1 (0–2)	<0.001^‡‡^
Mixed vegetables	3 (1–3)		2 (1–2)		2 (1–3)	0.03^†^
Meat, fish and eggs						
Minced meat Schnitzel	2 (1–3)		1 (1–2)		1 (0–2)	<0.001^‡‡^
Breaded chicken	2 (2–3)		2 (1–2)		1 (0–3)	0.004^‡^
Fish (white and oily)	1 (0–2)		2 (1–3)		2 (1–3)	<0.001*
Processed meat						
Hot dog	2 (1–3)		1.5 (0–2)		1 (0–2)	<0.001^‡‡^
Sausage	2 (1–3)		2 (1–2.5)		1 (0–1)	<0.001^‡‡^
Salami	3 (2–4)		2 (1–3)		1 (0–2)	<0.001^‡‡^
Pate	3 (1–3)		2 (1–3)		1 (1–2)	0.007^‡^
Bacon	3 (2–3)		1.5 (1–2.25)		1 (0–2)	<0.001^‡‡^
Bologna	2 (0–4)		1 (0–2)		1 (0–2)	<0.001^‡^
Salty snacks, sweets and cakes						
Crisps (any kind)	2 (1–3)		1 (0–3)		1 (0–1)	<0.001**
Biscuits	2 (1–3)		1 (0.5–2)		1 (0–1)	0.001**
Cakes (dry, creamy)	2 (1–3)		1 (0–2)		1 (0–1.5)	<0.001^‡‡^
Chocolate	3 (2–3)		2 (1–3)		1 (1–2)	<0.001^‡‡^
Chewing gum (with sugar, sugar-free)	3 (2–4)		2 (1–3)		2 (0.5–3)	0.001^‡^
Fat and oil						
Butter	3 (1–4)		2 (1–3)		2 (1–3)	0.02^‡^
Oil	4 (2–5)		3 (2–4)		3 (1–4)	0.001^§^
Fast food						
Hamburger	2 (1–3)		1 (0–2)		0 (0–1)	<0.001^‡‡^
Pizza	2 (1–3)		1 (0–2)		0 (0–1)	<0.001^‡‡^

^†^significantly more consumed at *t*=0 day than after *t*=30 day, ^‡^significantly most consumed at *t*=0 day, ^§^significantly less consumed after *t*=60 day than at *t*=0 day, *significantly least consumed at *t*=0 day, **significantly least consumed after *t*=60 day, ^‡‡^frequency of consumption is significantly different between all measurements, p<0.05

One of the limitations of this study is that the dietary habits in this study are specific to the population of eastern Croatia and may be very different from those of other regions, but nevertheless it shows a difference in the consumption of certain food groups between participants with and without LPR. Furthermore, FFQ-m questionnaire has so far been validated only for the adolescent population and not for adults. Another limitation of this study is that the treatment outcome was assessed using the subjective LPR-HRQL questionnaire, which is still a subjective measure, and that the quality of life was compared only between the use of esomeprazole and pantoprazole. In addition, this study could be further adapted and extended by including an additional dietary questionnaire and comparing the quality of life after the use of different proton pump inhibitors available on the market.

## CONCLUSIONS

Treatment with proton pump inhibitors at a dose of 20 mg twice daily and dietary and general lifestyle changes for a duration of two months reduced the symptoms across all domains of LPR and improved the quality of patients’ life. Patients with LPR consume more high-reflux potential foods and drink more carbonated beverages and juices than the healthy population, who consume more low-reflux potential foods and non-alcoholic beverages. Changing dietary habits to include low-reflux potential foods and water while avoiding acidic, spicy, fermented, sweet, fried foods, other high-reflux potential foods, carbonated beverages and juices significantly reduces symptoms in all domains of LPR and improves quality of life. The use of LPR-HRQL as an instrument can facilitate future research that aims to evaluate and compare different LPR therapies.
